# ITO nanoparticles reused from ITO scraps and their applications to sputtering target for transparent conductive electrode layer

**DOI:** 10.1186/s40580-017-0117-y

**Published:** 2017-09-06

**Authors:** Sung-Jei Hong, Sang-Hyun Song, Byeong Jun Kim, Jae-Yong Lee, Young-Sung Kim

**Affiliations:** 1 Display Materials and Components Research Center, Korea Electronics Technology Institute, Seongnam, South Korea; 2 Han Chung RF Co., Ltd, Incheon, South Korea; 3 Graduate School of Nano IT Design Fusion, Seoul National University of Science & Technology, Seoul, South Korea

**Keywords:** ITO-NPs, Reuse, Reverse reduction in situ dispersion, ITO target, TCE layer

## Abstract

In this study, ITO nanoparticles (ITO-NPs) were reused from ITO target scraps to synthesize low cost ITO-NPs and to apply to make sputtering target for transparent conductive electrodes (TCEs). By controlling heat-treatment temperature as 980 °C, we achieved reused ITO-NPs having Brunauer, Emmett and Teller specific surface area (BET SSA) and average particle size 8.05 m^2^/g and 103.8 nm, respectively. The BET SSA decreases along with increasing heat-treatment temperature. The ITO-NPs were grown as round mound shape, and highly crystallized to (222) preferred orientations. Also, applying the reused ITO-NPs, we achieved an ITO target of which density was 99.6%. Using the ITO target, we achieved high quality TCE layer of which sheet resistance and optical transmittance at 550 nm were 29.5 Ω/sq. and 82.3%. Thus, it was confirmed that the reused ITO-NPs was feasible to sputtering target for TCEs layer.

## Background

In recent, interests on fourth generation industrial revolution have been arisen, and it is strongly related to artificial intelligence (AI), internet of thing (IoT), advanced reality (AR), and virtual reality (VR), etc. It is heavily dictated by customer demand, and the customers intensively demand smaller and lighter electronics devices with high cost-effectiveness [1]. To fulfill the demand, materials for the devices have to be supplied easily to lower cost manufacturing. One of factors determining the cost of the devices is transparent conductive electrodes (TCEs), and indium tin oxide (ITO) is mainly used as the TCEs. ITO is consistent of tin (Sn) doped indium oxide (In_2_O_3_). The In is an important material for the ITO owing to its unique characteristics, high optical transparency and electrical conductivity. However, the In is being exhausted in the earth in proportion to growth of the related market (display, photovoltaics, lightings, touch sensors, etc.) [2, 3]. In general, In is recycled from redundant ITO target scraps that is emitted from the factories using them [4]. Because the In is recycled while being separated from Sn as indivisual [5], current process to manufacture ITO target increases processing steps that leads to higher manufacturing cost; i.e., In and Sn is indivisually oxidized to make In_2_O_3_ nanoparticles (NPs) and SnO_2_ NPs, and the two oxides are mixed together to dope Sn into In_2_O_3_ while sintering ITO target. Thus, improvement of recycling process to decrease steps and manufacturing cost is demanded for cost-effective devices [6]. One of improvement is to make targets with ITO-NPs instead of indivisual In_2_O_3_ NPs and SnO_2_ NPs and, therefore, ITO has to be reused from redundunt ITO sputtering target to synthesize in its form. In this study, an attempt to reuse ITO-NPs from redandant ITO target scraps is introduced to synthesize low cost ITO-NPs and to apply to make sputtering target for transparent conductive electrodes (TCEs).

## Methods

Firstly, ITO scraps were dissolved in a HCl with concentration of 100 g/L. As well, 0.1 wt% polyvinylpyrrolidones (PVPs), as a dispersing agent, were dissolved into NH_4_OH solution, as a reducing agent. Then, the HCl solution was put into the NH_4_OH solution to precipitate In-Sn hydroxide particles. The precipitates were washed several times until residuals such as NH^4+^ and Cl^−^ were erased out. The precipitate particles were heat-treated at 400, 600, 800 and 980 °C for 2.5 h to crystallize ITO-NPs, respectively. The specific surface area (SSA), crystal structure, particle size, composition ratio of the ITO-NPs were analyzed by means of a Brunauer, Emmett & Teller specific surface area (BET SSA) analyzer, X-ray diffractometer (XRD, Rigaku Rotaflex D/MAX System) with monochromatic Cu target (λ = 0.1541 nm), field emission scanning transmission electron microscope (FESEM, MZ-15/EC/JSM-7000F), high resolution transmission electron microscope (HRTEM, JEOL, JEM-3010) and Inductively coupled plasma (ICP) spectrometer (Thermo, iCAP 7000).

Then, a 2-in. sized ITO target was made using the ITO-NPs followed by sintering at 1580 °C for 15 h and ITO thin films were coated on a 3 × 3 cm^2^ sized glass substrate by sputtering. Microstructures of ITO target and ITO thin films were observed with FESEM and, to determine feasibilty of application of ITO-NPs to ITO target, their characteristics were evaluated by measuring electrical sheet resistance and optical transmittance with a four -point probe electrical measurement system (Mitsubishi Chemical Analytech, MCP-T610) and a UV–VIS Spectrophotometer (JASCO, V-560).

## Results and discussion

**Fig. 1:**
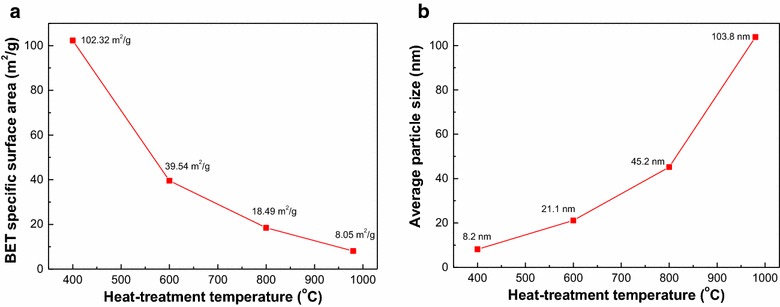
**a** BET SSA and **b** average particle size of ITO-NPs dependent on heat-treatment temperature

In Fig. 1a, BET SSAs of the ITO-NPs heat-treated at 400, 600, 800 and 980 °C were 102.32, 39.54, 18.49 and 8.05 m^2^/g respectively. The BET SSA decreased along with increasing heat-treatment temperature. Also, their average particle size was calculated from the BET SSA as follows;

1 $$ {\text{D }} = 6/\uprho \cdot {\text{d }} $$

where D, ρ and d are particle size, SSA and density, respectively. In Fig. 1b, the calculated average particle sizes of ITO-NPs heat-treated at each temperature were 8.2, 21.1, 45.2 and 103.8 nm, respectively. The particle size increased along with increasing heat-treatment temperature. The increase of particle size is owing to particle surface migration [7]. According to the transformation kinetics;

2 $$ {\text{D}} = {\text{D}}_{ 0} \cdot {\exp }\left( - {\text{E/kT}} \right) $$

where D, D_0_, E, k and T are diffusivity of particle surface, its initial diffusivity, activation energy for particle surface migration, Boltzmann constant and temperature, respectively. When the temperature becomes high at constant activation energy, the diffusivity of ITO-NPs surface becomes high as a function of their temperature. Accordingly, the ITO-NPs are grown when the heat-treatment temperature is raised, and their physical properties such as particle size can be simply controlled by changing the heat-treatment temperature.

We controlled BET SSA and particle size of ITO-NPs by changing heat-treatment temperature, and ITO-NPs heat-treated at 980 °C, of which BET SSA and particle size were 8.05 m^2^/g and 103.8 nm, was chosen to improve target density. The target density is an important factor that affects electrical and optical properties of ITO coating layer by sputtering method [8]. Lower resistivity and higher transparency can be obtained from the sputtering target with higher density and, in general, the higher density can be obtained with larger sized nanoparticles. The reason was mentioned at the later part of this section.

**Fig. 2:**
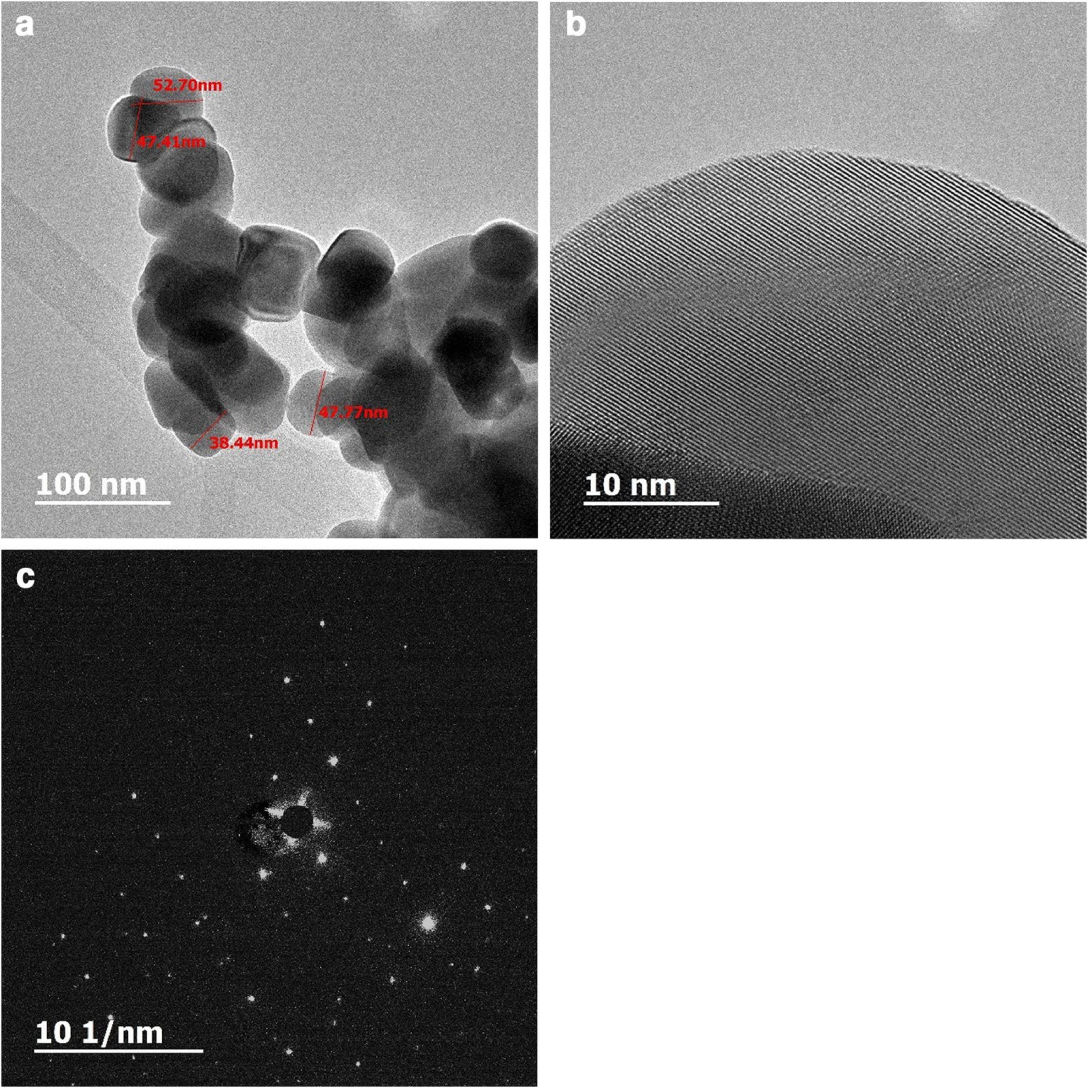
**a** HRTEM observation, **b** its magnification and **c** SAED pattern of ITO-NPs synthesized with reverse reduction in situ method and heat-treatment at 980 °C

**Fig. 3:**
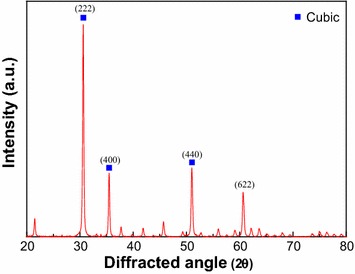
X-ray diffracted patterns of ITO-NPs heat-treated at 980 °C

Thus, we synthesized ITO-NPs and heat-treated at 980 °C. HRTEM observation of ITO-NPs heat-treated at 980 °C is shown in Fig. 2a. Primary particle sizes are c.a. 30–40 nm and they were agglomerated to exhibit larger size. In Fig. 2b morphology of the ITO-NPs are round mound shape and lattice structure were obviously observed. In fact, in Fig. 2c highly crystallized diffraction pattern was observed from SAED pattern. It is supposed that crystal structure of ITO-NPs was highly ordered to (222) preferred orientation while heat-treating at the high temperature, 980 °C. In fact, XRD analysis shows that they are crystalline ITO-NPs. In Fig. 3, all the detected peaks of the nanoparticle samples was corresponded with that of crystallized ITO. From the patterns, very intense peaks were observed at the three most important peaks of In_2_O_3_ namely 〈222〉, 〈400〉, 〈440〉 reflections. The peaks do not deviate from the PDF intensities, implying random non-oriented arrangement of the ITO-NPs. The major peaks due to SnO_2_ at 26.5° and SnO at 33.2° 2θ were absent in the observed pattern, indicating complete miscibility of In and Sn in the proposed composition [9]. It is known that each Sn^4+^ replaces In^3+^ in ITO lattice and, thereby, donating a free electron for the conductivity. Therefore, the ITO materials retain the cubic In_2_O_3_ structure up to the solid solubility limit of the SnO_2_ in In_2_O_3_ [10]. ICP analysis revealed that composition ratio of Into Sn of the reused ITO-NPs was 89.95–9.98 in weight. Also, full width half maximum (FWHM) of the peak was wider than that of the commercialized ITO particles. The result indicates that the size of the ITO-NPs is nanocrystal according to the Scherrer’s equation [11]. From the X-ray diffraction peak, particle size can be calculated by using Scherrer’s equation as;

3 $$ {\text{t}} = 0.9\uplambda / {\text{B cos}}\,\uptheta_{\text{B}} $$

where t, λ, B, and θ_B_ are particle size, wavelength (0.1542 nm for CuK_α_ radiation), FWHM of a peak in radians, and diffracted angle, respectively. In Eq. (3), the intensity peak increases along with a reduction in the peak half width indicating the growth of ITO-NPs. Accordingly, particle size becomes smaller as the FWHM is widened. Average particle size calculated from FWHM was c.a. 34.5 nm, which is almost in accordance with HRTEM observation in Fig. 2a. The average particle size, 103.8 nm, calculated from BET SSA, is attributed to agglomeration between particles by diffusion and particle growth under high temperature.

Nevertheless, smaller particle size shown in the HRTEM and FWHM is concerned with Derjaguin, Landau, Verwey and Overbeek (DVLO) theory [12]. In DLVO theory, the potential energy of van der Waals attraction and the potential energy of the electrical double layer interaction [13] are summed to provide a total interaction potential energy between colloidal particles. Due to differences in the surface chemistries of dispersing agent and ITO-NPs, obtaining a stable dispersion of polymeric dispersing agent and ITO-NPs can be challenging. The importance of the colloidal stability of starting dispersions on the final properties of ink pastes has been demonstrated. Zhao et al. [14] found that the presence of aggregated titanium dioxide particles in dispersions deleteriously affects the optical and mechanical properties. Researchers have found that the colloidal stability of dispersions can be disrupted by changes in surface potential of dispersing agent [15], ionic strength [15], concentration of dispersing agent [13] and particle size [13]. One way to address the questions about clustering and stability in ink paste is through predictions using DLVO theory [15]. Under drying above the latex glass transition temperature, particles consolidated and compacted, forcing the ITO-NPs to segregate into the boundary regions between dispersing agent, PVP.

**Fig. 4:**
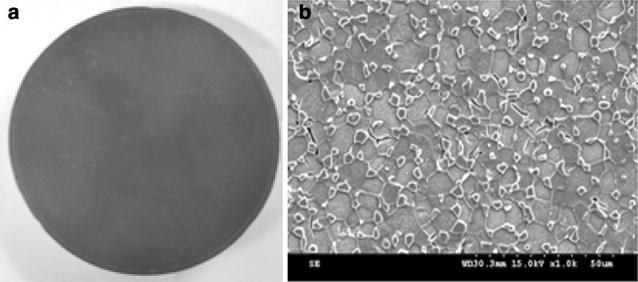
**a** Photograph and **b** SEM observation of sputtering target made of reused ITO-NPs heat-treated at 980 °C

Using the ITO-NPs, we fabricated ITO target. In Fig. 4a, 2-in. sized ITO target was well fabricated, and its microstructure was highly dense as shown in Fig. 4b. In fact, sintered density of the ITO target was measured to be 7.126 g/cm^3^. Considering that theoretical density of ITO is 7.155 g/cm^3^, we calculated as follows; (7.127/7.155) × 100 = 99.61%. That is, we got an ITO target with density of 99.6%. The value is very high enough to commercialization. It is attributed to using the ITO-NPs of which size was 103.8 nm, as mentioned earlier. In fact, we experienced that relatively lower value of density (87.9%) was obtained when smaller sized (21 nm) ITO-NPs was used to make a target. In contrary, target density was improved to 96% when we used larger sized (103.8 nm) ITO-NPs. Then, using the 103.8 nm sized ITO-NPs, we achieved ITO target of which density is 99.6% by optimizing the sintering conditions. It is reported that [16] the driving force of sintering leads to the reduction of total surface energy in system. Solid sintering can be divided into three stages initial-stage sintering, mid-stage sintering and final-stage sintering. The interfaces, namely “necks”, are formed among raw powders in the initial-stage sintering. Grain growth and pore connection occur simultaneously in the middle-stage sintering. In final-stage sintering, the pores become isolated while grain boundaries are linked each other and grains grow rapidly, so that the densification rate decreases evidently.” From the behavior, it is supposed that larger sized ITO particles reduce pore size by decreasing ITO grain growth rate leading to improvement of generation and isolation of pores. Thus, it is supposed that target density was enhanced by using larger-sized ITO-NPs.

**Fig. 5:**
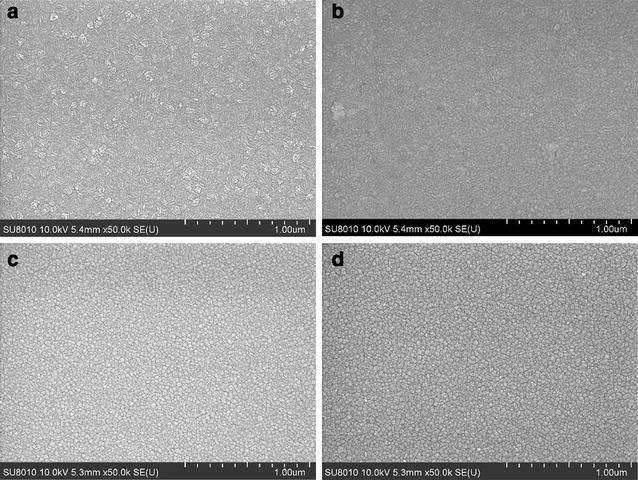
FESEM observations of ITO layers by sputtering with reused ITO target dependent on film thickness; **a** 130 nm, **b** 180 nm, **c** 250 nm, and **d** 350 nm

**Fig. 6:**
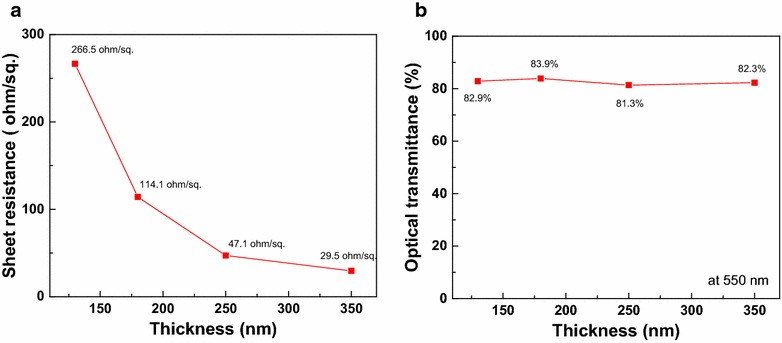
**a** Sheet resistance and **b** optical transmittance of ITO layers by sputtering with reused ITO target dependent on film thickness

The microstructures of ITO layers coated by using sputtering method are shown in Fig. 5a–d, respectively. Thickness of the ITO layers were 130, 180, 250, and 350 nm, respectively. It was observed that grain was grown when the thickness was raised. In Fig. 6a, their sheet resistances were 266.5, 114.1, 47.1, and 29.5 Ω/sq. respectively. The sheet resistance decreased as the film thickness increased. It is owing to thickness dependence of metallic layer [17]. The sheet resistance is determined using the simple equation;

4 $$ {\text{R}}_{\text{s}}  = \uprho / {\text{t}} $$

where R_s_, ρ, and t are sheet resistance, resistivity, and thickness, respectively. From the Eq. (4), assuming that the resistivity is constant, the sheet resistance is reversely proportional to film thickness. Whereas, in Fig. 6b, their optical transmittances at 550 nm (T_550_) were 82.9, 83.9, 81.3, and 82.3%, respectively. It is attributed to clearly coated layer from highly densified ITO target. Those T_550_ values are lower than that of commercial TCEs. We suggest that it is attributed to amorphous structured TCE layer just after deposition without heat-treatment. Thus, further work is to improve optical and electrical properties by enhancing physical properties of ITO film by, for example, optimizing heat-treatment conditions (temperature, environments), etc. Although further works are being done to improve ITO layers, ITO-NPs reused from ITO target scraps is feasible to apply to make sputtering target for TCEs.

## Conclusions

In this study, ITO-NPs were reused from redundant ITO target scraps to synthesize low cost ITO-NPs and to apply to make sputtering target for TCEs. By controlling heat-treatment temperature as 980 °C, we achieved reused ITO-NPs having BET SSA and average particle size 8.05 m^2^/g and 103.8 nm, respectively. The BET SSA decreases with raised heat-treatment temperature. The ITO-NPs were grown to round mound shape, and highly crystallized to (222) preferred orientations. Also, applying the reused ITO-NPs, ITO target, of which high density of 99.6%, was achieved. Using the ITO target, we achieved high quality TCE layer having sheet resistance and optical transmittance at 550 nm of 29.5 Ω/sq. and 82.3%. Thus, it was confirmed that the reused ITO-NPs was feasible to sputtering target for TCEs layer.
